# Temporal Trends in Hospitalization and Interventions for Aortic Stenosis in Canada, 2012-2024

**DOI:** 10.1016/j.cjco.2026.06.015

**Published:** 2026-07-01

**Authors:** Maria Servito, Denis Qeska, Suleman Aktaa, Philippe Pibarot, Jessica Forcillo, David Messika-Zeitoun, Janine Eckstein, Ellen Ross, Kendra MacFarlane, Seema Nagpal, Harindra C. Wijeysundera

**Affiliations:** a Division of Cardiac Surgery, University of Manitoba, Winnipeg, Manitoba, Canada; b Institute of Health Policy, Management, and Evaluation, University of Toronto, Toronto, Ontario, Canada; c Department of Medicine, University of British Columbia, Vancouver, British Columbia, Canada; d Centre for Heart Valve Innovation, St Paul's Hospital, Vancouver, British Columbia, Canada; e Department of Cardiology, Institut Universitaire de Cardiologie et de Pneumologie de Québec, Université Laval, Québec City, Québec, Canada; f Department of Cardiac Surgery, Centre Hospitalier de l'Université de Montréal, Montréal, Québec, Canada; g Division of Cardiology, University of Ottawa Heart Institute, Ottawa, Ontario, Canada; h Faculty of Medicine, School of Epidemiology and Public Health, University of Ottawa, Ottawa, Ontario, Canada; i Royal University Hospital, University of Saskatchewan, Saskatoon, Saskatchewan, Canada; j Heart Valve Voice Canada, Toronto, Ontario, Canada; k Canadian Cardiovascular Society, Ottawa, Ontario, Canada; l Division of Cardiology, Schulich Heart Program, Sunnybrook Health Sciences Centre, University of Toronto, Toronto, Ontario, Canada; m ICES, Toronto, Ontario, Canada

**Keywords:** aortic stenosis, surgical aortic valve replacement, transcatheter aortic valve implantation

## Abstract

**Background:**

Given the paradigm shift from surgical aortic valve replacement (SAVR) to transcatheter aortic valve implantation (TAVI) for severe aortic stenosis (AS), we examined the temporal trends in AS hospitalization and treatment across Canada.

**Methods:**

Data from the Canadian Institute for Health Information Discharge Abstract Database were used for a population-based cross-sectional study of inpatients between April 1, 2012, and March 31, 2024. We estimated national and provincial rates and reported the annual changes in hospitalization for AS, SAVR, and TAVI. Rates were expressed as episodes/procedures per million, and stratified by age group, sex, and urban-vs-rural residence.

**Results:**

Between 2012 and 2024, hospitalizations for AS increased from 259.8 to 362.4 per million. Rates of SAVR declined from 104.4 to 66.5 per million, whereas rates of TAVI rose from 19.0 to 136.9 per million. The average annual per-million increases in AS hospitalizations were highest in Prince Edward Island (25.0), Nova Scotia (22.9), and Newfoundland and Labrador (21.3), particularly among individuals ≥ 80 years of age. The rates of SAVR declined, as the uptake of TAVI increased, with the shift mostly evident in those 60-79 years of age. Rates of TAVI were higher in men than in women, whereas AS hospitalizations were higher in rural than in urban areas.

**Conclusions:**

Not unexpectedly there has been a dramatic shift from SAVR to TAVI observed, with an overall improvement in access to care. Nevertheless, sex-related and geographic disparities persist, highlighting the need for a coordinated national effort to ensure equitable access to AS management.

## Résumé

**Contexte:**

Compte tenu du changement de paradigme observé dans la prise en charge de la sténose aortique (SA) sévère, avec le passage de la chirurgie de remplacement valvulaire aortique à l’implantation valvulaire aortique par cathéter (IVAC), nous avons examiné les tendances temporelles des hospitalisations et des traitements liés à la SA à l’échelle du Canada.

**Méthodologie:**

Les données issues de la Base de données sur les congés des patients (BDCP) de l’Institut canadien d’information sur la santé (ICIS) ont été utilisées pour mener une étude populationnelle transversale, portant sur les patients hospitalisés entre le 1^er^ avril 2012 et le 31 mars 2024. Nous avons estimé les taux nationaux et provinciaux, et rapporté les variations annuelles des hospitalisations pour SA, chirurgie de remplacement valvulaire aortique et IVAC. Les taux ont été exprimés en nombre d’épisodes ou d’interventions par million d’habitants, et stratifiés par tranche d’âge, par sexe et selon le lieu de résidence (urbain ou rural).

**Résultats:**

Entre 2012 et 2024, les hospitalisations pour sténose aortique ont augmenté, passant de 259,8 à 362,4 par million. Les taux de chirurgie de remplacement valvulaire aortique ont diminué, passant de 104,4 à 66,5 par million, tandis que ceux d’IVAC ont augmenté, passant de 19,0 à 136,9 par million. Les augmentations annuelles moyennes par million des hospitalisations pour SA étaient les plus élevées à l’Île-du-Prince-Édouard (25,0), en Nouvelle-Écosse (22,9) et à Terre-Neuve-et-Labrador (21,3), en particulier dans le groupe des 80 ans et plus. Les taux de chirurgie de remplacement valvulaire aortique ont diminué à mesure que le recours à l’IVAC augmentait, cette évolution étant particulièrement marquée dans le groupe des 60 à 79 ans. Les taux d’IVAC étaient plus élevés chez les hommes que chez les femmes, tandis que les hospitalisations pour SA étaient plus nombreuses en milieu rural qu’en milieu urbain.

**Conclusion:**

Sans surprise, un déplacement marqué de la chirurgie de remplacement valvulaire aortique vers l’IVAC était observé, accompagné d’une amélioration globale de l’accès aux soins. Néanmoins, des disparités liées au sexe et à la situation géographique persistent, soulignant la nécessité d’un effort national coordonné pour garantir un accès équitable à la prise en charge de la SA.

Degenerative aortic stenosis (AS) is the most commonly acquired valvular heart disease in North America. Severe AS is strongly associated with poor prognosis, with a nearly 50% risk of mortality at 4 years if left untreated.^1^ Surgical aortic valve replacement (SAVR) has traditionally been the mainstay treatment for severe AS. Over the past decade and a half, transcatheter aortic valve implantation (TAVI) has transformed the treatment paradigm, becoming the predominant strategy in patients > 70 years of age.^2^ Temporal and geographic trend analyses on the burden and treatment of AS in the United States and Europe provided critical insights for health care resource allocation by illuminating disparities in care and guiding quality improvement interventions.^1^^,^^3^^,^^4^ In Canada, similar studies have been conducted for conditions such as congenital heart disease, atherosclerotic cardiovascular disease, and sudden cardiac death.^5^, ^6^, ^7^ However, no pan-Canadian analyses have evaluated trends in the burden and treatment strategies for AS. Therefore, we examined Canada-specific hospitalization rates for AS and assessed temporal trends in SAVR and TAVI across provinces/territories, stratified by rural-urban status and sex.

## Methods

### Data sources and study population

We conducted this cross-sectional study using inpatient records from April 1, 2012 to March 31, 2024 to evaluate the trends of hospitalization and treatment of AS. Data were obtained from the **C**anadian **I**nstitute for **H**ealth **I**nformation **D**ischarge **A**bstract **D**atabase (CIHI-DAD), which captures demographic, diagnostic, and procedural information for all patients discharged from acute inpatient facilities across all Canadian provinces and territories, with the exception of Québec. Given that the data were de-identified, the requirement for informed consent was waived. Institutional ethics review was conducted by CIHI as per standard protocols.

Age was categorized into 3 groups: 0-64 years, 65-79 years, and ≥ 80 years. Records were excluded if they represented duplicate entries, abandoned intervention cases, or contained missing or invalid age and/or sex information. Hospitalizations for AS were identified if any diagnosis column contained the relevant **I**nternational **C**lassification of **D**iseases (ICD) codes listed in Supplemental Table S1. Hospitalizations involving valve interventions were identified using **C**anadian **C**lassification of Health **I**nterventions (CCI) codes for SAVR and TAVI, as specified in Supplemental Table S1, if present in any of the 3 intervention columns. In cases where multiple diagnoses or interventions were recorded within a single hospitalization, each diagnosis and/or intervention was counted only once per hospitalization to avoid double counting. Annual rates for each diagnosis and intervention were calculated by fiscal year, defined as April 1 to March 31. Trends were further examined by age group, sex, and province. To evaluate geographic disparities, trends in rural and urban areas were evaluated within each province. Rural and urban regions were defined using patient postal codes, and were classified using the Statistical Area Classification type, which is the CIHI’s standard definition.

### Statistical analysis

Crude hospitalization rates were calculated by dividing counts within the fiscal year by the corresponding fiscal year population estimates obtained from Statistics Canada; these were subsequently expressed as rates per million.^8^^,^^9^ Due to the relatively small population, counts from Nunavut, the Northwest Territories, and Yukon were aggregated into a single group (“Territories”); for the Territories, data were only available for AS hospitalizations. Rate calculations were independently performed by 2 authors (D.Q. and M.S.), and consensus was achieved prior to analysis. Discrepancies were resolved by discussion and/or the senior author (H.W.).

Given that nonlinear temporal patterns were anticipated, trends over time were modeled using a generalized additive model. Average changes in hospitalization rates over the study period were quantified using the “marginaleffects” package in R version 4.3.1 (R Foundation for Statistical Computing, Vienna, Austria), calculated from the mean of the partial derivatives of the regression function for each rate between 2012 and 2024. Differences in trends between Canada and other provinces/territories were evaluated using 2 one-sided equivalence tests in the “marginaleffects” package, wherein an equivalence margin was defined as within 50% of the 2012 national crude rate specific to the condition or intervention being evaluated. In this analysis, if the *P* value was < 0.05, then the null hypothesis that the differences are large was rejected (ie, the trend is similar). Differences between rural and urban hospitalization rates in each province were also assessed using the same range with one-sided equivalence tests. All statistical analyses were conducted using R software and the packages “marginaleffects,” “dplyr,” “ggplot,” and “gtsummary.” Geographic variation in disease burden and procedural rates was presented visually as heatmaps that were also generated in R using the “canadianmaps” and “ggplot2” packages.

## Results

### National trends

Between 2012 and 2024, the national crude hospitalization rate for AS increased steadily from 259.8 to 362.4 per million (Supplemental Table S2), corresponding to an average annual increase of 8.6 per million (Table 1). There was a marked shift in treatment patterns toward TAVI (Fig. 1). Rates of SAVR declined from 104.4 to 66.5 per million, representing an average annual decrease of 3.1 per million (Table 1). Conversely, the rates of TAVI increased more than 7-fold, rising from 19.0 to 136.9 per million (Supplemental Table S2), representing an average annual increase of 9.8 per million. Although this resulted in a net increase of AS interventions of 6.7 per million yearly, the total rate of interventions has remained lower than the rate of AS hospitalizations (Supplemental Fig. S1).

**Table 1:** Average change of annual rate per million for aortic stenosis, surgical aortic valve replacement, and transcatheter aortic valve implantation between 2012 and 2024

Location	Aortic stenosis			SAVR and TAVI			SAVR			TAVI		
	Average change∗	95% CI	*P* value	Average change∗	95% CI	*P* value	Average change∗	95% CI	*P* value	Average change∗	95% CI	*P* value
Canada	8.6	7.5, 9.8	< 0.001	6.7	6.1, 7.4	< 0.001	−3.1	−3.7, −2.4	< 0.001	9.8	9.4, 10.3	< 0.001
AB	7.9	6.4, 9.5	< 0.001	5.6	4.7, 6.4	< 0.001	−1.8	−2.6, −1.1	< 0.001	7.4	6.8, 7.9	< 0.001
BC	6.1	4.4, 7.8	< 0.001	4.4	3.0, 5.8	< 0.001	−4.5	−6.0, −3.0	< 0.001	8.9	7.5, 10.3	< 0.001
MB	0.1	−3.1, 3.4	0.94	2.2	−0.2, 4.5	0.07	−8.5	−10.0, −7.1	< 0.001	10.7	9.4, 12.0	< 0.001
NB	17.7	10.1, 25.3	< 0.001	11.4	6.3, 16.5	< 0.001	−4.4	−7.4, −1.4	< 0.001	15.7	13.1, 18.4	< 0.001
NL	21.3	14.5, 14.5	< 0.001	18.5	13.9, 23.0	< 0.001	3.6	−0.3, 7.4	0.07	14.9	13.56, 16.2	< 0.001
NS	22.9	18.2, 27.7	< 0.001	13.9	10.1, 17.7	< 0.001	−6.1	−9.3, −2.8	< 0.001	20.0	17.6, 22.3	< 0.001
ON	12.4	10.4, 14.4	< 0.001	10.6	9.2, 12.0	< 0.001	−4.3	−5.4, −3.2	< 0.001	14.9	14.0, 15.8	< 0.001
PE	25.0	20.0, 30.1	< 0.001	1.0	0.5, 1.4	< 0.001	NA	NA	NA	1.0	0.5, 1.4	< 0.001
SK	7.9	3.7, 12.1	< 0.001	8.0	5.8, 10.2	< 0.001	−5.3	−7.5, −3.1	< 0.001	13.3	11.7, 14.8	< 0.001
Territories	6.6	2.5, 11.0	< 0.001	NA	NA	NA	NA	NA	NA	NA	NA	< 0.001

AB, Alberta; BC, British Columbia; CI, confidence interval; MB, Manitoba; NB, New Brunswick; NL, Newfoundland and Labrador; NA, not available; NS, Nova Scotia; ON, Ontario; PE, Prince.

Edward Island; SK, Saskatchewan; SAVR, surgical aortic valve replacement; TAVI, transcatheter aortic valve implantation.

∗ Expressed as rate per 1,000,000.

**Figure 1:**
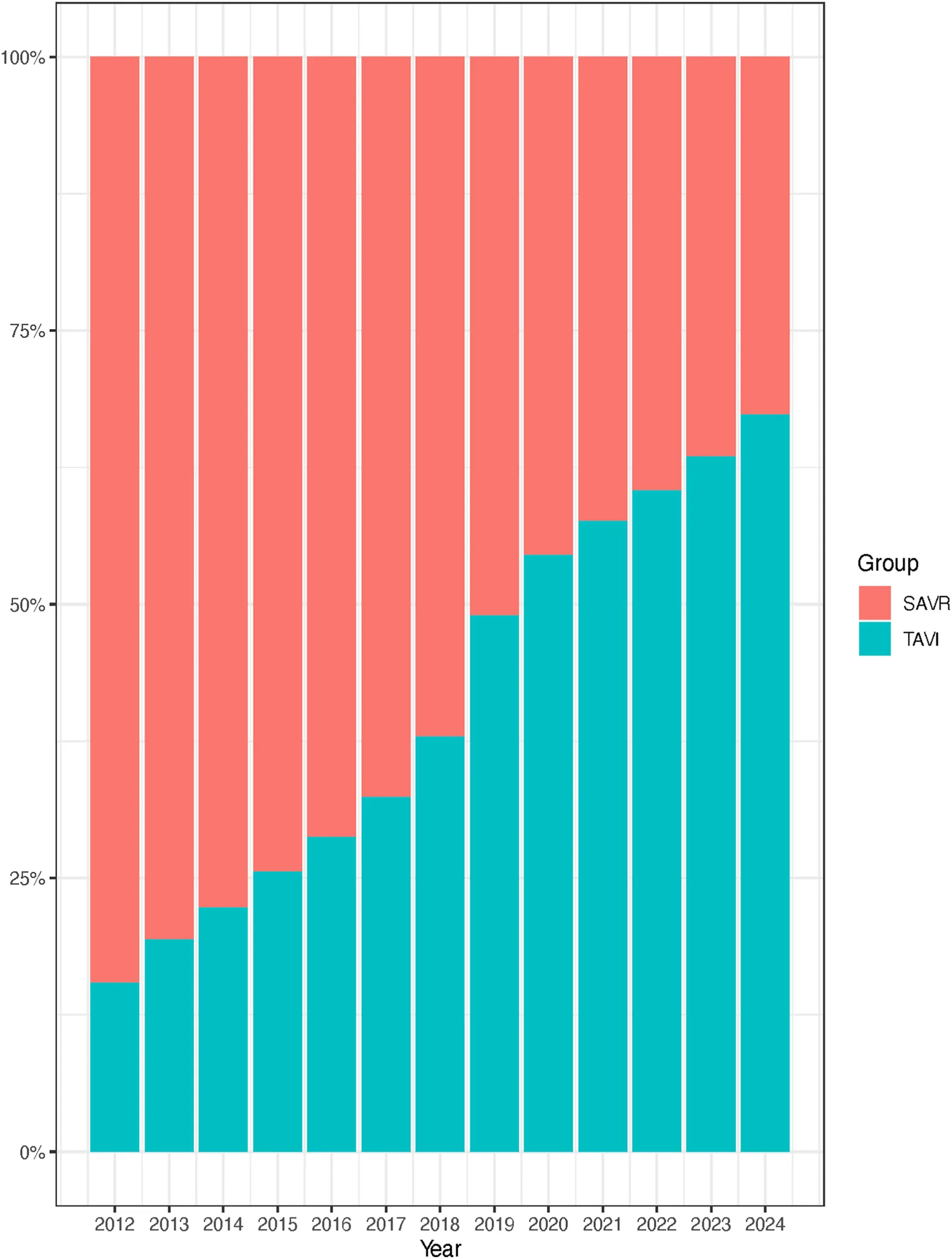
Bar charts showing the relative proportion of surgical aortic valve replacement (SAVR) and transcatheter aortic valve intervention (TAVI) for aortic stenosis between 2012 and 2024.

### Interprovincial variation

Interprovincial variation in hospitalizations for AS was observed (Fig. 2). The most pronounced increase in AS hospitalizations was observed in the Atlantic provinces; Prince Edward Island (25.0 per million), Nova Scotia (22.9 per million), and Newfoundland and Labrador (21.3 per million) exhibited annual increases of nearly 3-fold the national average (8.6 per million), whereas western provinces showed more modest growth (Table 1). Consequently, in 2024, the highest rates of AS hospitalization were observed in New Brunswick (730.5 per million) and Nova Scotia (731.5 per million), double the national rates of AS hospitalization (Table 2).

**Figure 2:**
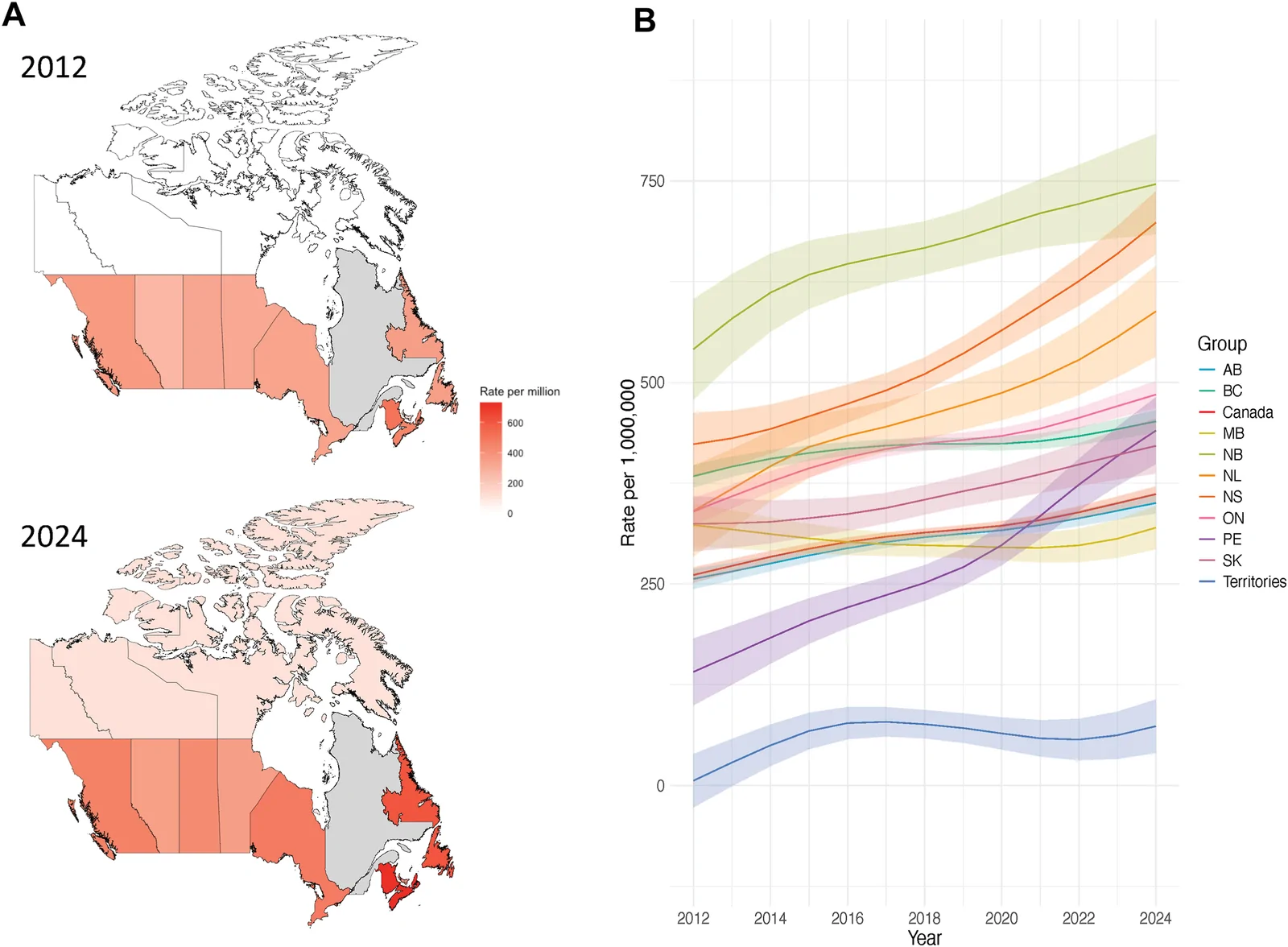
Temporal trend of hospitalization for aortic stenosis between 2012 and 2024. (**A**) Heatmaps elucidating the prevalence of hospitalization for aortic stenosis between in 2012 and 2014. (**B**) Rates of hospitalization for aortic stenosis according to province, expressed as rate per million. The bands indicate 95% confidence interval. AB, Alberta; BC, British Columbia; MB, Manitoba; NB, New Brunswick; NL, Newfoundland and Labrador; NS, Nova Scotia; ON, Ontario; PI, Prince Edward Island; SK, Saskatchewan.

**Table 2:** Results of test of equivalence using 2 one-sided test to determine whether the difference in rates for aortic stenosis, surgical aortic valve replacement, and transcatheter aortic valve implantation, between other provinces/territories and Canadian rates is large

Location	*P* values∗		
	AS	SAVR	TAVI
AB	< 0.001	< 0.001	1.00
BC	< 0.001	< 0.001	1.00
MB	< 0.001	< 0.001	1.00
NB	1.00	0.99	0.43
NL	0.94	0.99	1.00
NS	1.00	1.00	0.07
ON	< 0.001	0.93	1.00
PE	< 0.001	< 0.001	1.00
SK	< 0.001	1.00	1.00
Territories	1.00	0.00	0.95

AB, Alberta; AS, aortic stenosis; BC, British Columbia; CI, confidence interval; MB, Manitoba; NB, New Brunswick; NL, Newfoundland and Labrador; NS, Nova Scotia; ON, Ontario; PE, Prince Edward Island; per million, per million population; SAVR, surgical aortic valve replacement; SK, Saskatchewan; TAVI, transcatheter aortic valve implantation.

∗ Test of equivalence, where the null hypothesis is that the difference between the Canadian rate and provincial rate is large (ie., > 50% of the 2012 national crude rate).

Across all provinces, SAVR declined as TAVI increased substantially (Fig. 3). The largest reductions in SAVR rates were observed in Manitoba (−8.5 per million) and Nova Scotia (−6.1 per million). Of note, there was an increase, albeit not significant, in SAVR rates in Newfoundland and Labrador between 2012 and 2024 (Table 1). In 2024, the rate of TAVI was highest in Nova Scotia (267.8 per million), New Brunswick (228.4 per million), and Ontario (198.0 per million), nearly twice the national rate (136.9 per million). The pace of TAVI growth also varied between provinces: the fastest expansions of TAVI occurred in Nova Scotia (20.0 per million), New Brunswick (15.7 per million), and Ontario (14.9 per million; Table 1), double the national annual average growth rate (9.8 per million). Only Alberta, British Columbia, and Manitoba had lower average annual TAVI rates than the national average, and this was consistent across age groups (Table 2).

**Figure 3:**
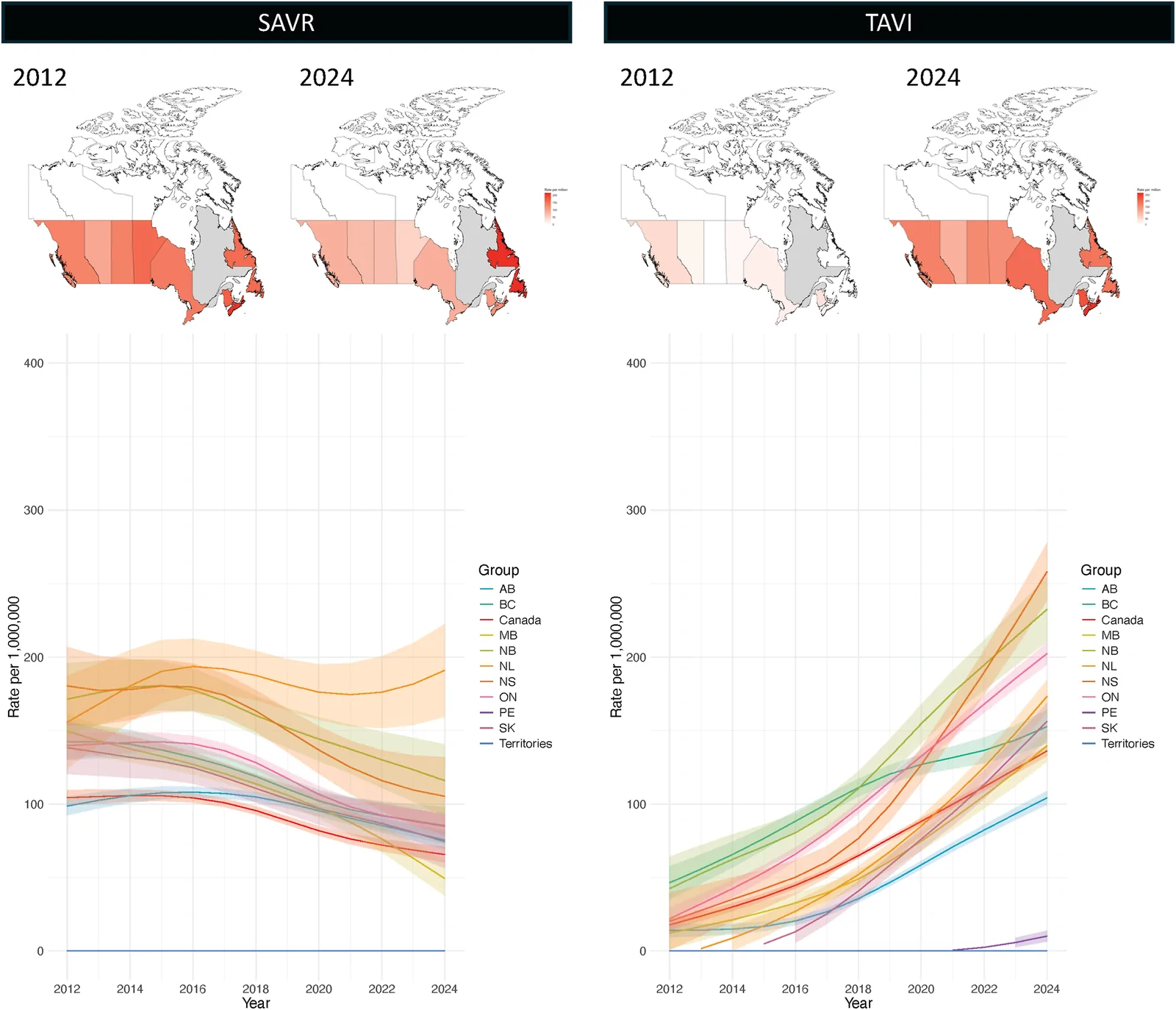
Temporal trend of interventions for aortic stenosis between 2012 and 2024. (**A**) Heatmaps and line graph illustrating the temporal trends of surgical aortic valve replacement (SAVR) between 2012 and 2024. (**B**) Heatmaps and line graph illustrating the temporal trends of transcatheter aortic valve implantation (TAVI) between 2012 and 2024. The bands indicate 95% confidence interval. AB, Alberta; BC, British Columbia; MB, Manitoba; NB, New Brunswick; NL, Newfoundland and Labrador; NS, Nova Scotia; ON, Ontario; PI, Prince Edward Island; SK, Saskatchewan.

### Demographic drivers

Age-specific analyses demonstrated that the growth in hospitalization for AS was driven predominantly by patients ≥ 80 years old, peaking at nearly 8000 per million among men in 2024 (Supplemental Fig. S2). Nationally, AS hospitalizations in the ≥ 80 years age group grew by 85.3 per million annually in men and 33.5 per million in women (Table 3). The rate of AS hospitalizations was similarly high in the 65-79 years age group, reaching nearly 3000 per million in men in 2024 (Supplemental Fig. S2). Notably, the fastest growth occurred in Prince Edward Island, with an average increase of 430.20 per million annually among men aged ≥ 80 years old. In contrast, the national rates of AS remained relatively low and stable among those in the < 65 years group.

**Table 3:** Average change in annual rate per million for hospitalizations for aortic stenosis by age

Location	Men with aortic stenosis			Women with aortic stenosis		
	Average change∗	95% CI	*P* value	Average change∗	95% CI	*P* value
< 65 years						
Canada	0.3	−0.1, 0.7	0.14	0.0	−0.3, 0.3	0.92
AB	0.1	−0.6, 0.8	0.76	0.6	−0.2, 1.3	0.16
BC	0.0	−1.0, 1.0	0.97	−0.5	−1.1, 0.2	0.15
MB	−3.9	−5.9, −1.9	< 0.001	-1.3	−3.0, 0.4	0.13
NB	1.4	−4.7, 7.5	0.65	1.1	−1.8, 4.1	0.45
NL	1.3	−3.0, 5.6	0.57	1.5	−2.0, 5.0	0.40
NS	5.6	3.4, 7.7	< 0.001	0.9	−1.7, 3.4	0.52
ON	0.5	−0.3, 1.2	0.24	−0.1	−0.5, 0.3	0.64
PE	−1.6	−5.2, 2.1	0.40	3.6	2.2, 4.9	< 0.001
SK	−0.4	−2.7, 2.0	0.77	−0.8	−2.4, 0.7	0.30
Territories	1.5	−2.3, 5.2	0.44	−0.3	−2.6, 2.0	0.81
65-79 years						
Canada	15.4	8.6, 22.2	< 0.001	8.3	4.9, 11.7	< 0.001
AB	0.6	−15.9, 17.0	0.94	−0.3	−9.5, 8.8	0.94
BC	−3.5	−13.6, 6.7	0.51	-0.9	−9.6, 7.7	0.84
MB	−40.1	−70.4, −9.9	0.01	0.8	−21.6, 23.2	0.94
NB	14.6	−23.4, 52.6	0.45	32.3	9.2, 55.4	0.01
NL	67.9	13.2, 122.6	0.02	26.7	3.3, 50.1	0.03
NS	59.1	23.7, 94.5	< 0.001	39.3	15.0, 63.6	< 0.001
ON	31.4	17.4, 45.4	< 0.001	11.9	5.7, 18.1	< 0.001
PE	110.3	72.1, 148.6	< 0.001	45.2	15.9, 74.6	< 0.001
SK	16.7	−13.0, 46.4	0.27	0.1	−23.0, 23.1	1.00
Territories	10.9	−23.0, 44.9	0.53	−9.9	−72.9, 53.1	0.76
≥ 80 years						
Canada	85.3	62.5, 108.0	< 0.001	33.5	19.3, 47.7	< 0.001
AB	137.4	86.3, 188.5	< 0.001	31.2	3.7, 58.7	0.03
BC	24.2	−15.5, 63.9	0.23	7.2	−13.9, 28.3	0.50
MB	122.7	49.0, 196.4	< 0.001	0.2	−32.8, 33.1	0.99
NB	83.7	−0.7, 168.1	0.05	−14.7	−127.4, 98.1	0.80
NL	79.6	−32.1, 191.2	0.16	26.9	−14.7, 68.4	0.21
NS	53.9	−72.6, 180.4	0.40	104.1	48.1, 160.2	< 0.001
ON	146.8	110.9, 180.4	< 0.001	57.9	35.4, 80.5	< 0.001
PE	430.2	239.1, 621.3	< 0.001	99.4	−53.4, 252.2	0.20
SK	162.3	115.2, 209.4	< 0.001	61.7	6.2, 117.1	0.03
Territories	119.5	−140.9, 379.9	0.37	192.5	−51.3, 436.3	0.12

AB, Alberta; BC, British Columbia; CI, confidence interval; MB, Manitoba; NB, New Brunswick; NL, Newfoundland and Labrador; NS, Nova Scotia; ON, Ontario; PE, Prince Edward Island; per million, per million population; SK, Saskatchewan.

∗ Expressed as rate per 1,000,000.

The transition of dominance from SAVR to TAVI was evident during the study period, and this shift was most pronounced in the 65-79 years age group (Supplemental Figs. S3 and S4). Nearly all provinces showed a downward trend in SAVR in the 65-79 years age group (Supplemental Fig. S3); Manitoba had the steepest decline in SAVR rates (−64.2 per million annually; Supplemental Table S3). The average annual increase in TAVI rates in Manitoba was 52.14 per million, which did not fully offset the decline in SAVR rates (Supplemental Table S4). A declining pattern in the rates of SAVR was also observed in men in the ≥ 80 years age group (Supplemental Fig. S3), as TAVI grew rapidly in this group (Supplemental Fig. S4). However, when comparing men and women, the annual average increase in men was twice that of women (148.8 vs 74.0 per million). Conversely, SAVR rates in the < 65 years age group remained stable, with rates ranging between 25 and 50 per million (Supplemental Fig. S3).

### Rural and urban trends

Nationally, AS hospitalization rates increased more rapidly in residents from rural than urban areas, with an average annual growth of 12.1 vs 8.0 per million, respectively (Supplemental Fig. S5). Although the national rates of AS hospitalization were not significantly different between rural and urban regions, province-specific AS hospitalization rates from rural areas in Alberta, Saskatchewan, Ontario, Nova Scotia, Prince Edward Island, and Newfoundland and Labrador were higher than in urban areas (Supplemental Table S5). Rates of intervention were also higher in rural vs urban areas (Supplemental Table S5).

## Discussion

In this population-based cross-sectional study, we examined the temporal and geographic trends of AS hospitalizations, SAVR, and TAVI, along with geographic, age-based, and sex-specific variation in these trends. Our study findings can be summarized as follows: 1) we observed a growing burden of AS, particularly among individuals 65-79 and ≥ 80 years of age; 2) there has been a marked increase in TAVI, supplanting SAVR as the primary AS intervention across all provinces; 3) the pace of TAVI growth varied by province; 4) similar patterns of variation were observed by sex, with higher rates of intervention in men than in women; and 5) hospitalizations for AS were higher in rural than in urban areas.

The rising hospitalization rates for AS we observed are consistent with prior population-based investigations, which appear largely attributable to the aging population. Multiple studies have demonstrated that the burden of clinically significant AS increases steeply with age, particularly among individuals ≥ 80 years of age, reflecting progressive calcific and degenerative etiologies.^10^, ^11^, ^12^ Our findings reinforce concerns that AS may represent the next major cardiovascular epidemic, particularly in high-income countries with rapidly aging populations. The growing burden of AS carries substantial economic implications for the Canadian health care system. Prior work has estimated that hospitalizations for AS accounted for approximately $393 million in hospital costs during the 2014-2015 fiscal year.^13^ As the Canadian health care system is constrained by capacity and cost pressures, continued growth in AS hospitalizations is likely to exacerbate this current financial and resource strain. Strategies to curb avoidable hospitalizations for AS, including earlier diagnosis, improved surveillance strategies, and streamlined referral pathways, will therefore be increasingly important.

Our findings also demonstrate a notable transition in trends of SAVR to TAVI, particularly in the 65-79 years age group, representing a key transitional cohort in which SAVR is progressively replaced by TAVI. Historically, SAVR was the primary treatment modality for AS. However, TAVI has increasingly become the dominant intervention, offering a less invasive alternative for all risk groups, especially for elderly patients and those with high surgical risk. This shift toward TAVI is similar to the dominance of TAVI observed in the United States and reflects the findings from pivotal trials showing noninferiority of TAVI compared with SAVR in low-surgical-risk patients.^14^^,^^15^ The evidence from these trials have subsequently led the 2020 **A**merican **H**eart **A**ssociation (AHA) guidelines to recommend TAVI in patients ≥ 65 years old with low surgical risk.^2^^,^^16^^,^^17^ However, unlike the United States, where use of TAVI has expanded even among patients < 65 years old despite not being recommended in the guidelines,^18^ TAVI rates in the < 65 years age group in Canada have remained relatively low and stable over time, reflecting a more balanced and conservative approach, similar to the Europeans.^19^

Our findings also highlight a sex-related difference in the receipt of TAVI. Nationally and across individual provinces, men underwent TAVI at higher rates than women. Although the limited granularity of our data precludes inferences regarding sex-based disparities in access to TAVI, this pattern raises concern for potential inequity. In our cohort, hospitalizations for AS were more frequent among men, which may partially explain the higher rates of TAVI observed in this group. However, older epidemiologic studies have suggested that, among individuals > 80 years old, AS is more prevalent in women than in men.^20^^,^^21^ As evidenced by prior studies and the Electronic Physician Notification to Facilitate the Recognition and Management of Severe Aortic Stenosis (DETECT AS) trial, women are underdiagnosed, given that they are more likely to present with paradoxical low-flow, low-gradient AS, which results in underestimation of disease severity and thus reduced referral for intervention.^22^, ^23^, ^24^, ^25^, ^26^ Indeed, studies have identified sex-related differences in access to and referral for TAVI, with women less likely to undergo transcatheter intervention and more likely to experience longer delays to intervention compared with men.^23^^,^^26^, ^27^, ^28^ Furthermore, women have a higher risk of mortality and procedural complications after SAVR.^29^

Disparities were also evident between residents of rural vs urban populations. Hospitalization rates for AS were consistently higher among residents from rural communities and increased more rapidly over time compared with urban areas. These findings may be due to the long-standing disparities faced by rural populations, which include reduced access to preventive care, delayed diagnosis, and limited availability of specialist and integrated valve care services. In such settings, hospitalization often serves as the first point of entry into the health care system, resulting from delayed diagnosis, later stage presentations, and higher inpatient utilization.^30^, ^31^, ^32^ This contrasts with individuals in urban areas, who are more likely to have access to integrated valve care, which can mitigate urgent hospitalizations due to AS.

Furthermore, our findings illustrate the growing mismatch between the increasing burden of AS and the capacity of the Canadian health care system to deliver timely interventions. In this study, hospitalizations attributable to AS increased over time; however, these estimates reflect inpatient encounters only and do not capture individuals diagnosed or managed in the outpatient setting, thereby underestimating the true population burden of AS. During the study period, rates of SAVR declined, whereas the overall rate of valve interventions rose, driven largely by the increased uptake of TAVI. Nevertheless, this growth in procedural volume has not kept pace with the rising burden of AS. Similar patterns have been observed across other priority areas of care in Canada, where procedural volumes have increased but waitlists continued to grow.^33^ Without improvements in system efficiency and capacity, delays in valve intervention are likely to worsen as the population continues to age. Jurisdiction-wide strategies, including timely adoption of innovative technologies, optimization of care pathways, early screening and detection, and expanded use of telehealth to support rural communities, may be necessary to increase procedural capacity while reducing late presentations and avoidable hospitalizations.

### Limitations

This study has several limitations. The cross-sectional design allowed for observations of trends only and precludes causal inference. Although this was an effort to capture pan-Canadian trends, Québec data were not analyzed because we only accessed CIHI-DAD for our study. Data from Québec are instead directly submitted to the CIHI by the ministère de la Santé et des Services sociaux du Québec, which is then appended to CIHI-DAD to create a separate database (Hospital Morbidity Database), and this was not accessed in this study. The use of administrative data introduces potential coding misclassification, and baseline patient characteristics and socioeconomic status were not available. The use of CIHI-DAD also limits granularity in the data, such as severity of AS and presence of symptoms. This highlights the need for a population-based data set that captures such details on AS for both outpatient and inpatient settings. Finally, in-hospital and longer term mortality data were not available within this data set. As such, the analysis of procedural outcomes after TAVI and SAVR falls outside the scope of this study, which was specifically designed to evaluate population-level hospitalization trends.

## Conclusions

In Canada, the burden of AS is growing, driven largely by adults aged ≥ 80 years old. Furthermore, TAVI has replaced SAVR as the predominant treatment strategy for AS in the elderly population. However, significant interprovincial, rural-urban, sex-related disparities in AS management persist. Specifically, sex-related differences suggest that women may experience underdiagnosis, lower rates of referral, and reduced access to interventions compared with men, despite comparable or greater symptom burden and adverse outcomes. These disparities may reflect a combination of biologic differences in disease presentation, sex-based biases in clinical assessment, and structural barriers within health care delivery. Collectively, our findings also point to an underprovision of interventions that has not kept pace with the growing burden of AS. This underscores an urgent need for efficient, integrated care pathways and timely adoption of innovative therapies to address the growing clinical and economic impact of valve disease, given the growing burden of AS attributed to the aging population in Canada. By providing province-specific and rural-vs-urban trend data, our findings move beyond a national diagnosis and offer a granular call to action. Because health care delivery in Canada falls under provincial jurisdiction, this evidence is positioned to directly inform provincial capacity planning, resource allocation, and the equitable expansion of aortic valve intervention to the populations and regions where unmet need is greatest. Future work should build on these trends by examining long-term post-intervention survival stratified by age and intervention type, particularly in older and extreme age groups. These analyses would further clarify whether current practice patterns reflect deliberate, survival-informed selection or are driven primarily by technical feasibility.

## Ethics Statement

The data utilized in this study were provided by the Canadian Institute for Health Information (CIHI). In accordance with CIHI's Privacy Policy and data sharing agreements, all data were fully de-identified prior to researcher access to prevent individual re-identification. Cell suppression rules were strictly applied to reporting any patient frequencies under five to protect individual and community confidentiality.

## Patient Consent

The authors confirm that patient consent is not applicable to this article. Given that the data were de-identified, the requirement for informed consent was waived.

## Funding Sources

This study was funded by the 10.13039/100013500Canadian Cardiovascular Society Heart Valve Working Group. Dr Wijeysundera is supported by a Canada Research Chair in Structural Heart Disease Policy and Outcomes.

## Disclosures

Dr Pibarot has received institutional funding from Edwards Lifesciences, Medtronic, Pi-Cardia, Cardiac Success, Boston Scientific, and Novartis for echocardiography core laboratory analyses and research studies in the field of valvular heart disease, for which he received no personal compensation. Dr Forcillo reports serving as a proctor, speaker, and consultant for Medtronic and Edwards Lifesciences, and as a consultant for Terumo. Dr Messika-Zeitoun has received a research grant from Edwards Lifesciences. The remaining authors have no conflicts of interest to disclose.
