# Supplementary material for: Temporal Trends in Hospitalization and Interventions for Aortic Stenosis in Canada, 2012-2024
Source: CJC Open. 2026 Jul 1;8(10):1365–74. doi: 10.1016/j.cjco.2026.06.015 (PMC13654382; doi:10.1016/j.cjco.2026.06.015)

Supplementary Material

Table of Contents

Table S1. International Classification of Disease – 10 Codes and Canadian Classification of Health Intervention Codes. *3*

Table S2. Crude rates per million of aortic stenosis, surgical aortic valve replacement, and transcatheter aortic valve implantation, nationally and for each province. *4*

Table S3. Average change in annual rate per million population for SAVR by age. *6*

Table S4. Average change in annual rate per million population for TAVI by age. *7*

Table S5. Average annual change in rate per million of aortic stenosis, surgical aortic valve replacement, and transcatheter aortic valve implantation, according to regional area. *8*

Figure S1. Growth in the rates of aortic stenosis, surgical aortic valve replacement, and transcatheter aortic valve implantation, by province. *9*

Figure S2. Aortic stenosis by age and sex. *10*

Figure S3. Surgical aortic valve replacement by age and sex. *11*

Figure S4. Transcatheter aortic valve implantation by age and sex. *12*

Figure S5. Regional variation in aortic stenosis, surgical aortic valve replacement, and transcatheter aortic valve implantation. *13*

Supplemental Table S1. International Classification of Disease – 10 Codes and Canadian Classification of Health Intervention Codes.

**Diagnosis**	**ICD-10-CA Code**	**ICD-10-CA Code Description**	**Diagnosis Type**
Aortic Stenosis	I35.0	Aortic (valve) stenosis	M, 1, 2, 6, W, X, Y
	I35.2	Aortic (valve) stenosis with insufficiency	M, 1, 2, 6, W, X, Y
**Diagnosis**	**CCI Code**	**CCI Code Description**	
Transcatheter aortic valve replacement	1HV90STXXL, 1HV80STXXL	Percutaneous transluminal (transcatheter) arterial or transfemoral:	
	1HV90STXXL, 1HV80STXXL	Transapical/transaortical/axillary (i.e. Non-transfemoral):	
	1HV90GRXXL, 1HV80GRXXL	Percutaneous transluminal (transcatheter) (transseptal) venous or Other (transseptal):	
Surgical aortic valve replacement	1HV90GRXXL, 1HV80GRXXL	Percutaneous transluminal (transcatheter) (transseptal) venous or Other (transseptal):	

Notes: CCI, Canadian Classification of Health Interventions; ICD-10-CA, International Classification of Disease -10-Canada.

Supplemental Table S2. Crude rates per million of aortic stenosis, surgical aortic valve replacement, and transcatheter aortic valve implantation, nationally and for each province.

Province	2012	2013	2014	2015	2016	2017	2018	2019	2020	2021	2022	2023	2024
Aortic stenosis													
AB	262.7	251.4	279.6	285.8	299.6	296.4	320.6	319.8	296.8	321.1	331.7	347.2	349.6
BC	371.5	404.1	417.4	403.3	417.8	419.1	439.0	431.4	403.0	424.8	429.4	445.3	456.5
MB	319.4	327.7	308.4	307.7	296.8	295.9	290.5	326.3	285.5	299.6	293.6	261.5	356.5
NB	509.2	585.6	643.2	667.8	613.1	676.9	637.3	665.1	682.9	794.1	629.3	788.1	730.5
NL	353.5	320.7	382.6	526.2	406.0	394.5	478.8	494.7	463.1	519.9	498.8	553.1	607.1
NS	450.6	403.3	409.5	499.2	484.6	483.1	480.2	546.2	564.1	620.1	625.0	609.7	731.5
ON	338.6	352.3	378.1	397.8	410.3	417.1	442.7	434.8	404.8	429.9	461.4	486.5	480.3
PE	145.4	152.8	159.6	255.8	197.4	267.1	236.4	269.6	238.7	345.4	406.7	431.7	417.3
SK	326.9	344.9	277.0	376.9	329.4	312.0	371.9	388.2	351.2	385.4	403.8	414.0	418.3
Territories	0.0	26.0	25.7	101.4	99.9	73.9	48.7	96.4	71.5	47.0	46.5	22.9	111.7
Canada	259.8	269.2	283.9	299.2	302.6	306.1	323.5	324.3	303.9	326.2	338.1	355.6	362.4
Surgical aortic valve replacement													
AB	95.3	101.5	110.3	108.4	107.7	102.0	116.3	100.8	89.9	87.3	86.2	83.8	74.0
BC	132.6	148.9	152.4	129.7	128.0	124.0	139.4	101.6	94.5	89.7	94.2	95.0	84.1
MB	158.5	127.4	139.3	136.1	129.4	122.1	105.7	116.8	86.2	92.0	88.4	55.1	47.6
NB	168.8	167.5	180.6	187.0	195.2	180.0	127.2	164.7	127.6	163.1	103.9	143.2	109.5
NL	157.7	155.6	155.3	244.2	188.8	177.4	206.3	180.1	153.7	184.0	176.9	152.2	215.8
NS	206.8	137.3	157.8	210.2	179.2	194.3	164.2	138.4	139.5	116.0	116.2	97.8	118.2
ON	141.7	134.3	139.1	143.4	144.3	143.5	140.7	114.5	94.6	90.9	90.6	94.1	86.9
PE	0.0	0.0	0.0	0.0	0.0	0.0	0.0	0.0	0.0	0.0	0.0	0.0	0.0
SK	135.7	148.3	103.4	141.7	143.6	106.3	114.2	103.9	79.7	98.5	95.0	81.0	71.3

Supplemental Table S2. Crude rates per million of aortic stenosis, surgical aortic valve replacement, and transcatheter aortic valve implantation, nationally and for each province.

Province	2012	2013	2014	2015	2016	2017	2018	2019	2020	2021	2022	2023	2024
Territories	0.0	0.0	0.0	0.0	0.0	0.0	0.0	0.0	0.0	0.0	0.0	0.0	0.0
Canada	104.4	101.8	105.0	107.5	106.0	103.1	104.0	86.8	73.9	73.0	71.9	71.7	66.5
Transcatheter aortic valve implantation													
AB	95.3	101.5	110.3	108.4	107.7	102.0	116.3	100.8	89.9	87.3	86.2	83.8	74.0
BC	132.6	148.9	152.4	129.7	128.0	124.0	139.4	101.6	94.5	89.7	94.2	95.0	84.1
MB	158.5	127.4	139.3	136.1	129.4	122.1	105.7	116.8	86.2	92.0	88.4	55.1	47.6
NB	168.8	167.5	180.6	187.0	195.2	180.0	127.2	164.7	127.6	163.1	103.9	143.2	109.5
NL	157.7	155.6	155.3	244.2	188.8	177.4	206.3	180.1	153.7	184.0	176.9	152.2	215.8
NS	206.8	137.3	157.8	210.2	179.2	194.3	164.2	138.4	139.5	116.0	116.2	97.8	118.2
ON	141.7	134.3	139.1	143.4	144.3	143.5	140.7	114.5	94.6	90.9	90.6	94.1	86.9
PE	0.0	0.0	0.0	0.0	0.0	0.0	0.0	0.0	0.0	0.0	0.0	0.0	0.0
SK	135.7	148.3	103.4	141.7	143.6	106.3	114.2	103.9	79.7	98.5	95.0	81.0	71.3
Territories	0.0	0.0	0.0	0.0	0.0	0.0	0.0	0.0	0.0	0.0	0.0	0.0	0.0
Canada	104.4	101.8	105.0	107.5	106.0	103.1	104.0	86.8	73.9	73.0	71.9	71.7	66.5

Notes: *95%CI,* 95% confidence interval; *AB,* Alberta; *BC,* British Columbia; *MB,* Manitoba; *NB,* New Brunswick; *NL,* Newfoundland and Labrador; *NS,* Nova Scotia; *ON,* Ontario; *PE,* Prince Edward Island; *SK,* Saskatchewan.

Supplemental Table S3. Average change in annual rate per million population for SAVR by age.

Location	SAVR in Males			SAVR in Females		
	Average Change[a]	(95%CI)	p-value	Average Change[a]	(95%CI)	p-value
**<65 years**						
Canada	-0.3	(-0.6, -0.03)	0.03	-0.2	(-0.4, -0.1)	<0.001
AB	0.0	(-0.6, 0.6)	0.94	0.1	(-0.3, 0.5)	0.56
BC	-0.4	(-1.2, 0.5)	0.43	-0.5	(-1.0, 0.02)	0.06
MB	-3.5	(-4.6, -2.4)	<0.001	-1.6	(-3.0, -0.3)	0.02
NB	0.6	(-3.0, 4.1)	0.75	0.4	(-1.7, 2.5)	0.70
NL	1.7	(-1.6, 5.0)	0.31	1.7	(-0.4, 3.7)	0.11
NS	0.6	(-1.2, 2.3)	0.54	0.1	(-1.6, 1.9)	0.88
ON	-0.5	(-1.0, 0.04)	0.07	-0.4	(-0.6, -0.2)	<0.001
PE	0.0	NA	NA	0.0	NA	NA
SK	-0.8	(-2.2, 0.6)	0.24	-0.9	(-1.8, -0.03)	0.04
Territories	0.0	NA	NA	0.0	NA	NA
**65-79 years**						
Canada	-24.5	(-29.4, -19.7)	<0.001	-13.2	(-15.6, -10.5)	<0.001
AB	-29.1	(-39.0, -19.2)	<0.001	-13.9	(-19.6, -8.2)	<0.001
BC	-31.2	(-42.0, -20.4)	<0.001	-17.9	(-24.4, -11.4)	<0.001
MB	-64.2	(-82.0, -46.4)	<0.001	-23.4	(-32.6, -14.3)	<0.001
NB	-62.5	(-81.1, -43.9)	<0.001	-6.8	(-15.3, 1.8)	<0.001
NL	6.8	(-24.3, 37.9)	0.67	-9.1	(-21.9, 3.7)	0.16
NS	-45.1	(-69.4, -20.7)	<0.001	-26.1	(-38.8, -13.3)	<0.001
ON	-30.2	(-39.5, -20.9)	<0.001	-19.0	(-23.4, -14.6)	<0.001
PE	NA	NA	NA	NA	NA	NA
SK	-52.6	(-76.0, -29.3)	<0.001	-23.5	(-32.9, -14.1)	<0.001
Territories	NA	NA	NA	NA	NA	NA
**≥80 years**						
Canada	-62.0	(-68.7, -55.3)	<0.001	-27.6	(-30.2, -25.0)	<0.001
AB	-69.5	(-83.4, -55.5)	<0.001	-24.0	(-31.0, -17.1)	<0.001
BC	-87.9	(-95.5, -80.4)	<0.001	-35.9	(-43.5, -28.2)	<0.001
MB	-90.2	(-120.9, -59.5)	<0.001	-45.3	(-54.5, -36.1)	<0.001
NB	-79.1	(-112.6, -45.6)	<0.001	-22.5	(-36.8, -8.2)	<0.001
NL	-12.9	(-32.5, 6.6)	0.19	-6.3	(-19.0, 6.4)	0.33
NS	-110.0	(-140.7, -79.3)	<0.001	-41.6	(-58.2, -24.9)	<0.001
ON	-83.6	(-93.9, -73.2)	<0.001	-42.0	(-46.8, -37.2)	<0.001
PE	NA	NA	NA	0.0	NA	NA
SK	-51.9	(-70.0, -33.8)	<0.001	-28.4	(-42.8, -13.9)	<0.001
Territories	NA	NA	NA	0.0	NA	NA

Notes: *95%CI,* 95% confidence interval; *AB,* Alberta; *BC,* British Columbia; *MB,* Manitoba; *NB,* New Brunswick; *NL,* Newfoundland and Labrador; *NS,* Nova Scotia; *ON,* Ontario; *PE,* Prince Edward Island; *SK,* Saskatchewan; *SAVR,* surgical aortic valve replacement. [a]*Unit: Rate per 1,000, 000*

Supplemental Table S4. Average change in annual rate per million population for TAVI by age.

Location	TAVI in Males			TAVI in Females		
	Average Change[a]	(95%CI)	p-value	Average Change[a]	(95%CI)	p-value
**<65 years**						
Canada	0.5	(0.4, 0.5)	<0.001	0.2	(0.2, 0.3)	<0.001
AB	0.2	(0.1, 0.3)	<0.001	0.1	(0.1, 0.2)	0.06
BC	0.1	(-0.2, 0.3)	0.60	0.0	(-0.1, 0.1)	0.84
MB	0.4	(0.1, 0.7)	<0.001	0.0	(-0.3, 0.3)	0.97
NB	0.5	(-0.7, 1.6)	0.42	0.8	(0.2, 1.5)	0.02
NL	1.4	(0.5, 2.3)	<0.001	1.1	(0.3, 2.0)	0.01
NS	2.1	(1.1, 3.0)	<0.001	1.0	(0.7, 1.2)	<0.001
ON	0.8	(0.7, 0.9)	<0.001	0.3	(0.3, 0.4)	<0.001
PE	NA	NA	NA	NA	NA	NA
SK	0.5	(0.2, 0.8)	<0.001	0.3	(-0.1, 0.7)	0.11
Territories	NA	NA	NA	NA	NA	NA
**65-79 years**						
Canada	39.4	(37.8, 41.1)	<0.001	22.5	(20.6, 24.4)	<0.001
AB	32.4	(27.9, 36.9)	<0.001	24.5	(20.9, 28.2)	<0.001
BC	36.8	(31.4, 42.2)	<0.001	16.8	(12.3, 21.2)	<0.001
MB	52.1	(43.5, 60.8)	<0.001	27.8	(22.4, 33.2)	<0.001
NB	73.7	(59.0, 88.4)	<0.001	41.0	(30.4, 51.6)	<0.001
NL	57.8	(46.7, 68.8)	<0.001	34.3	(27.5, 41.1)	<0.001
NS	89.1	(82.4, 95.9)	<0.001	47.4	(37.2, 57.6)	<0.001
ON	59.6	(55.4, 63.9)	<0.001	33.6	(29.8, 37.4)	<0.001
PE	NA	NA	NA	NA	NA	NA
SK	53.8	(42.0, 65.6)	<0.001	31.6	(26.7, 36.4)	<0.001
Territories	NA	NA	NA	NA	NA	NA
**≥80 years**						
Canada	148.8	(134.8, 162.8)	<0.001	74.0	(70.2, 77.8)	<0.001
AB	150.7	(126.8, 174.7)	<0.001	80.4	(70.0, 90.7)	<0.001
BC	117.6	(81.8, 153.5)	<0.001	62.4	(48.1, 76.7)	<0.001
MB	189.6	(150.2, 229.1)	<0.001	96.9	(67.9, 125.8)	<0.001
NB	149.9	(107.5, 192.3)	<0.001	39.9	(17.0, 62.8)	<0.001
NL	160.5	(116.7, 204.3)	<0.001	57.0	(43.4, 70.6)	<0.001
NS	202.2	(163.0, 241.3)	<0.001	89.4	(55.6, 123.2)	<0.001
ON	238.0	(219.8, 256.1)	<0.001	120.7	(110.4, 130.9)	<0.001
PE	52.3	(27.9, 76.7)	<0.001	NA	NA	NA
SK	272.5	(225.4, 319.5)	<0.001	105.5	(85.4, 125.6)	<0.001
Territories	NA	NA	NA	NA	NA	NA

Notes: *95%CI,* 95% confidence interval; *AB,* Alberta; *BC,* British Columbia; *MB,* Manitoba; *NB,* New Brunswick; *NL,* Newfoundland and Labrador; *NS,* Nova Scotia; *ON,* Ontario; *PE,* Prince Edward Island; *SK,* Saskatchewan; *TAVI,* transcatheter aortic valve implantation. [a]*Unit: Rate per 1,000, 000*

Supplemental Table S5. Average annual change in rate per million of aortic stenosis, surgical aortic valve replacement, and transcatheter aortic valve implantation, according to regional area.

Location	Urban			Rural			TOST p-value[a]
	Average Change[b]	(95%CI)	p-value	Average Change[a]	(95%CI)	p-value	
Aortic stenosis							
Canada	8.0	(7.0 9.1)	<0.001	12.1	(10.4, 13.9)	<0.001	<0.001
AB	8.3	(6.5, 10.0)	<0.001	9.2	(5.6, 12.9)	<0.001	0.08
BC	5.2	(3.5, 6.9)	<0.001	12.6	(7.0, 18.3)	<0.001	<0.001
MB	-0.7	(-4.6, 3.1)	0.72	2.4	(-1.4, 6.3)	0.21	<0.001
NB	17.3	(8.3, 26.3)	<0.001	20.1	(11.0, 29.3)	<0.001	<0.001
NL	16.4	(10.8, 21.9)	<0.001	32.5	(20.5, 44.5)	<0.001	0.33
NS	20.8	(16.0, 25.5)	<0.001	30.1	(20.3, 39.8)	<0.001	0.51
ON	11.6	(9.7, 13.5)	<0.001	20.6	(16.3, 24.8)	<0.001	0.91
PE	15.1	(8.0, 22.2)	<0.001	23.0	(14.5, 31.5)	<0.001	0.71
SK	8.5	(3.6, 13.4)	<0.001	8.6	(2.6, 14.5)	0.01	<0.001
Territories	14.4	(3.8, 24.9)	0.01	12.3	(4.1, 20.5)	<0.001	0.05
Surgical aortic valve replacement							
Canada	-3.0	(-3.6, -2.4)	<0.001	-2.8	(-3.7, -1.9)	<0.001	0.19
AB	-1.5	(-2.5, -0.5)	<0.001	-2.7	(-4.1, -1.3)	<0.001	1.00
BC	-4.5	(-6.3, -2.7)	<0.001	-2.4	(-4.3, -0.6)	0.01	0.93
MB	-7.9	(-9.6, -6.1)	<0.001	-8.9	(-12.5, -5.3)	<0.001	0.27
NB	-4.9	(-7.5, -2.2)	<0.001	-2.1	(-5.8, 1.6)	0.26	0.15
NL	3.3	(-0.4, 7.0)	0.08	4.3	(-1.4, 10.0)	0.14	0.94
NS	-4.8	(-8.2, -1.5)	<0.001	-6.7	(-12.0, -1.4)	0.01	0.98
ON	-4.4	(-5.4, -3.4)	<0.001	-3.6	(-6.2, -1.0)	0.01	1.00
PE	NA	NA	NA	NA	NA	NA	NA
SK	-3.6	(-6.5, -0.8)	0.01	-7.8	(-10.4, -5.1)	<0.001	0.33
Territories	NA	NA	NA	NA	NA	NA	NA
Transcatheter aortic valve implantation							
Canada	9.7	(9.3, 10.2)	<0.001	12.0	(11.2, 12.8)	<0.001	0.87
AB	7.3	(6.7, 7.9)	<0.001	9.6	(8.5, 10.8)	<0.001	1.00
BC	10.4	(9.0, 11.8)	<0.001	10.8	(9.0, 12.6)	<0.001	0.99
MB	10.3	(8.7, 11.9)	<0.001	10.9	(9.0, 12.7)	<0.001	1.00
NB	14.6	(11.8, 17.4)	<0.001	17.4	(13.8, 21.0)	<0.001	1.00
NL	13.4	(11.7, 15.0)	<0.001	17.6	(16.0, 19.1)	<0.001	0.97
NS	15.6	(12.5, 18.6)	<0.001	26.7	(23.1, 30.3)	<0.001	1.00
ON	14.4	(13.4, 15.4)	<0.001	21.6	(19.9, 23.3)	<0.001	1.00
PE	1.5	(0.8, 2.2)	<0.001	2.7	(1.4, 3.9)	<0.001	0.02
SK	12.8	(11.2, 14.4)	<0.001	15.2	(12.5, 17.9)	<0.001	0.97
Territories	NA	NA	NA	NA	NA	NA	NA

Notes: *95%CI,* 95% confidence interval; *AB,* Alberta; *BC,* British Columbia; *MB,* Manitoba; *NB,* New Brunswick; *NL,* Newfoundland and Labrador; *NS,* Nova Scotia; *ON,* Ontario; *PE,* Prince Edward Island; *SK,* Saskatchewan;
[a]Test of equivalence, where the null hypothesis is that the difference in the average rates is large (i.e., > 50% of the 2012 national crude rate).
[b]*Unit: Rate per 1,000, 000*

Supplemental Figure S1. Growth in the rates of aortic stenosis, surgical aortic valve replacement, and transcatheter aortic valve implantation, by province.

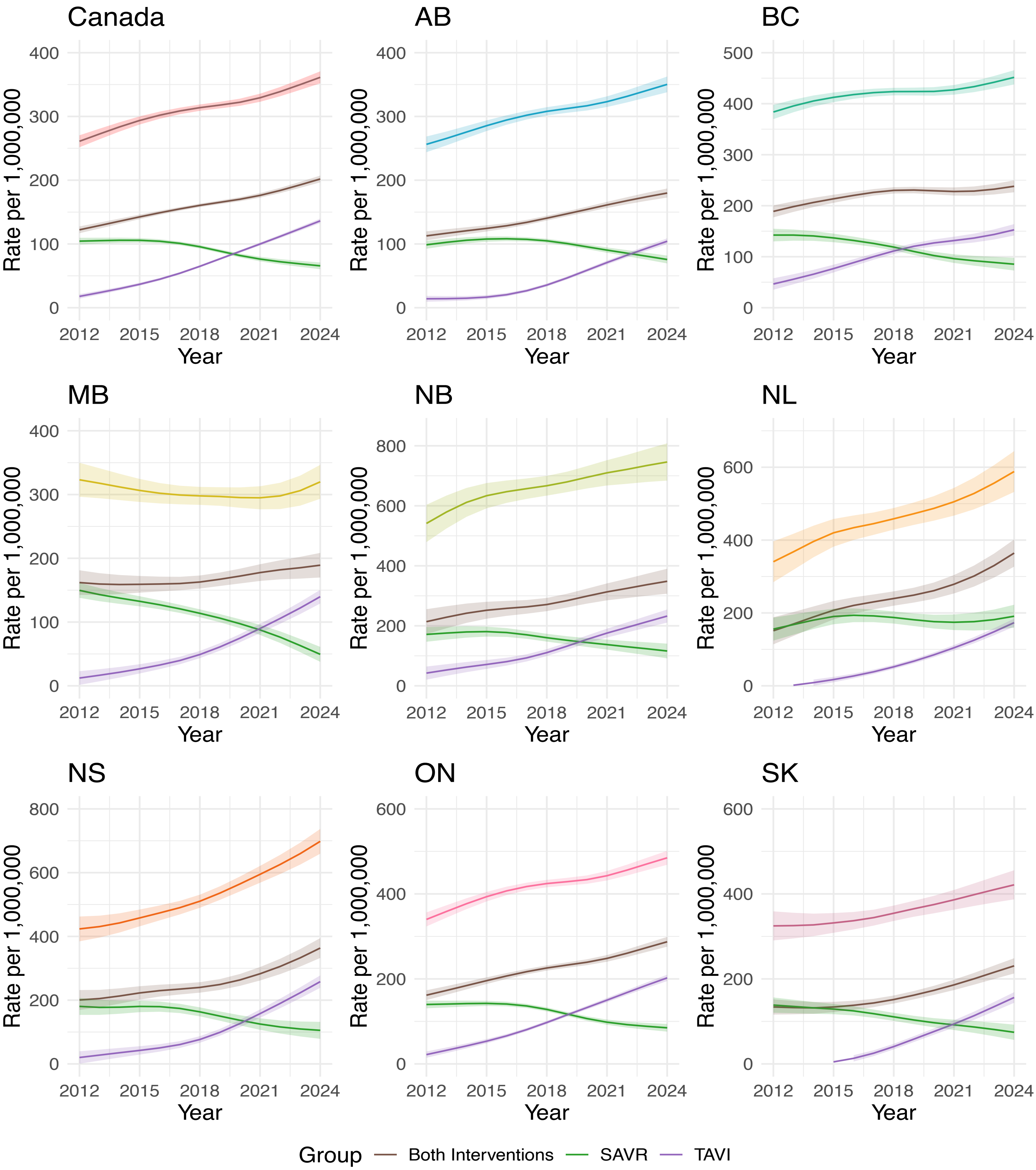

Supplemental Figure S2. Aortic stenosis by age and sex.

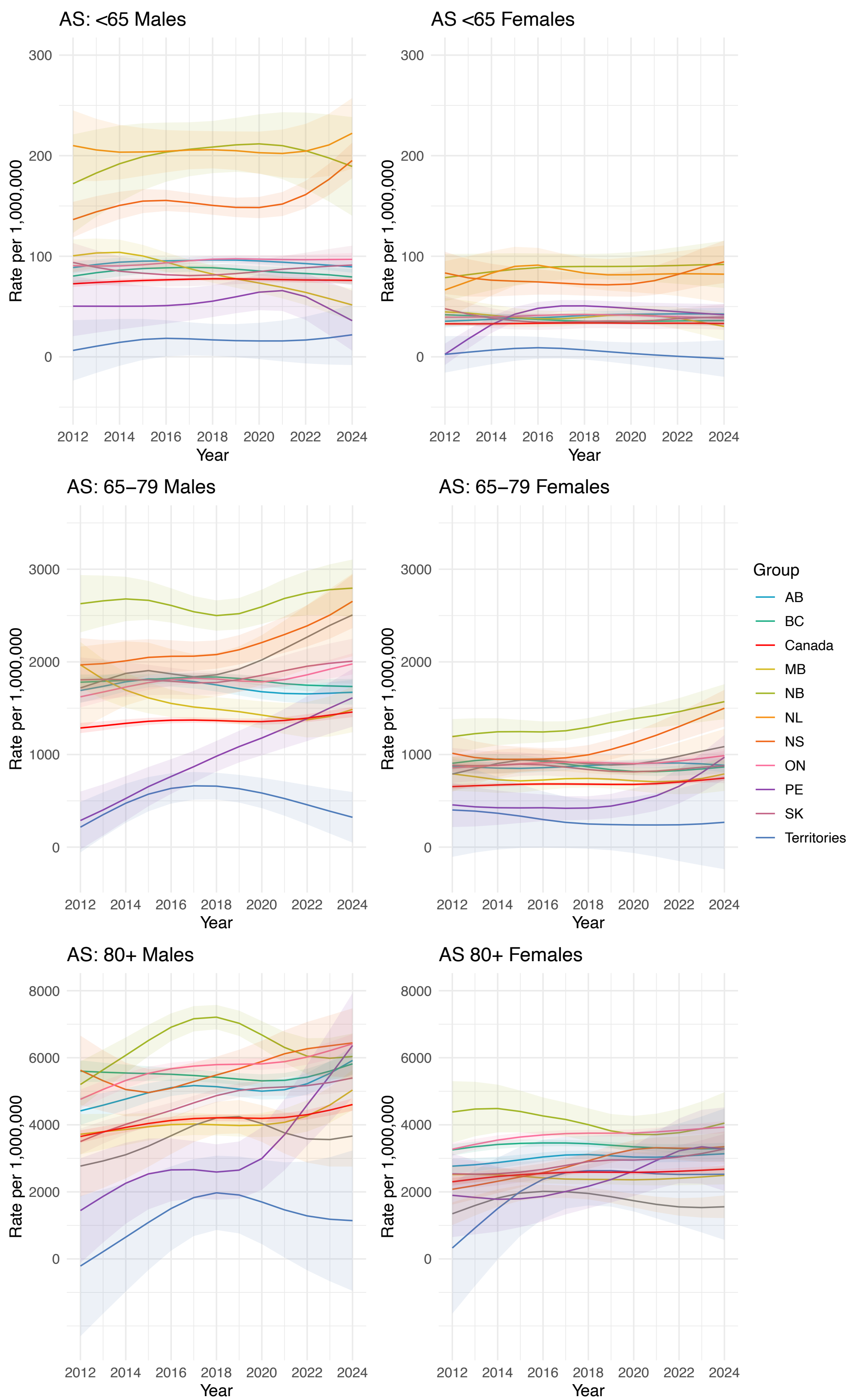

Supplemental Figure S3. Surgical aortic valve replacement by age and sex.

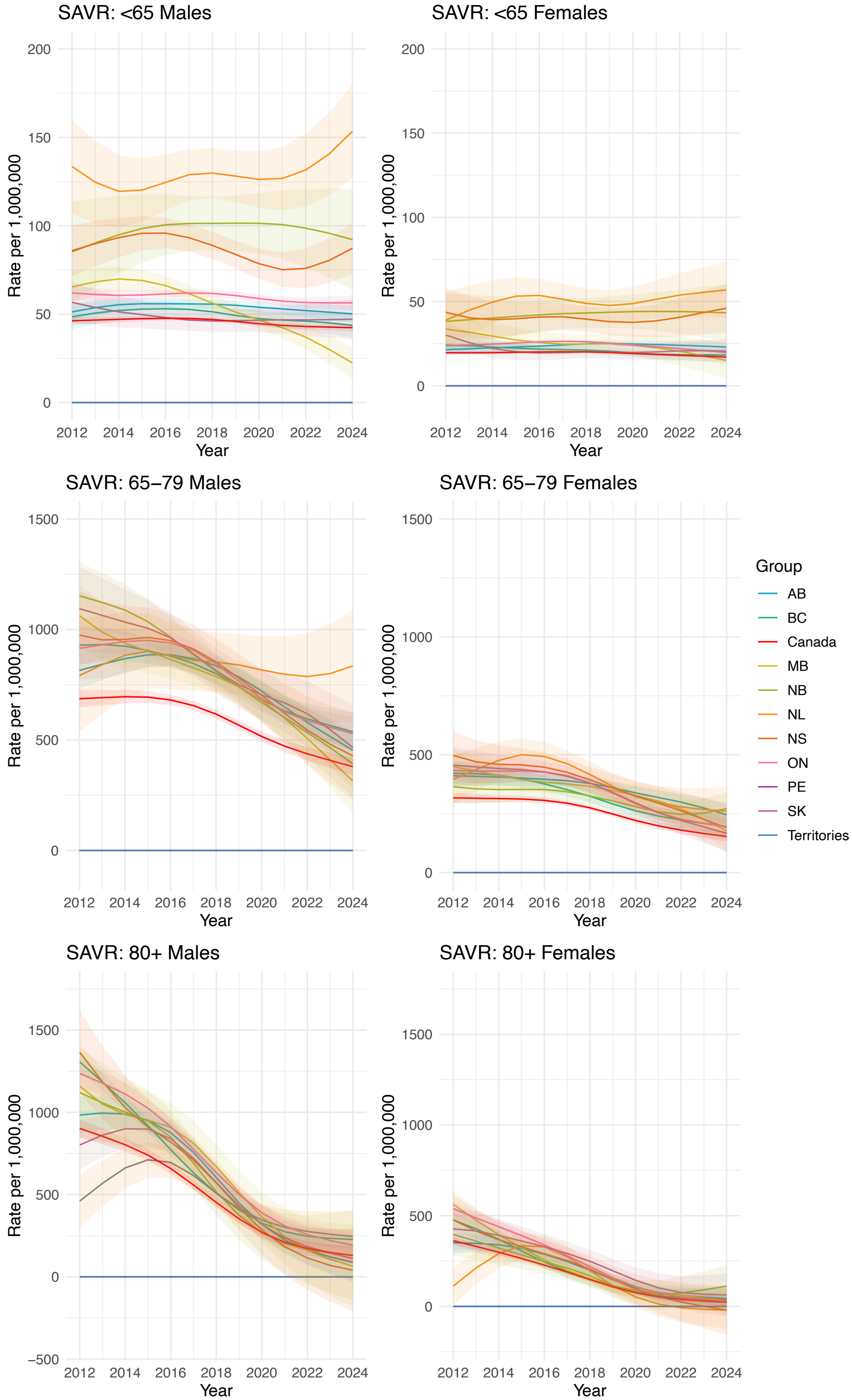

Supplemental Figure S4. Transcatheter aortic valve implantation by age and sex.

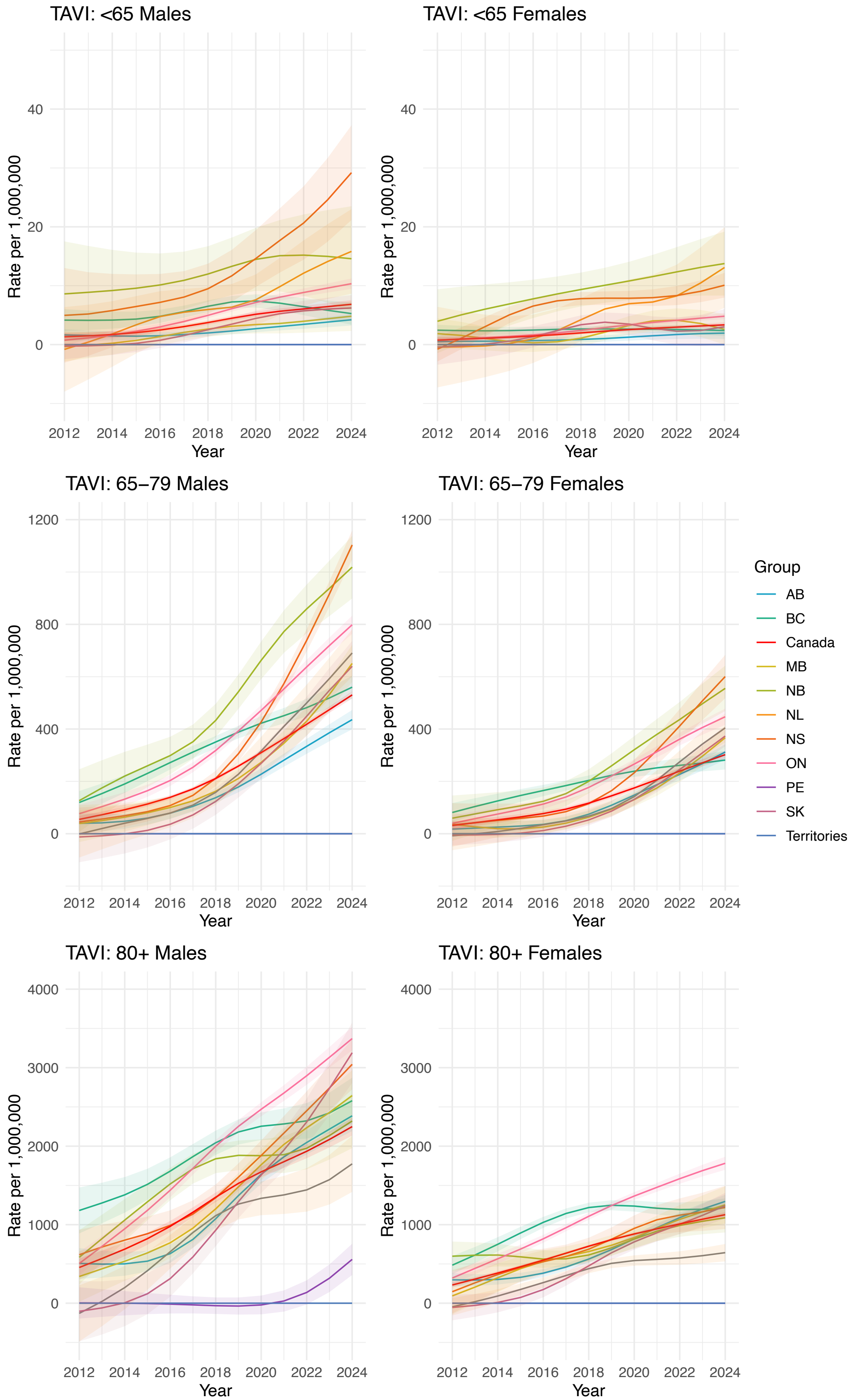

Supplemental Figure S5. Regional variation in aortic stenosis, surgical aortic valve replacement, and transcatheter aortic valve implantation.

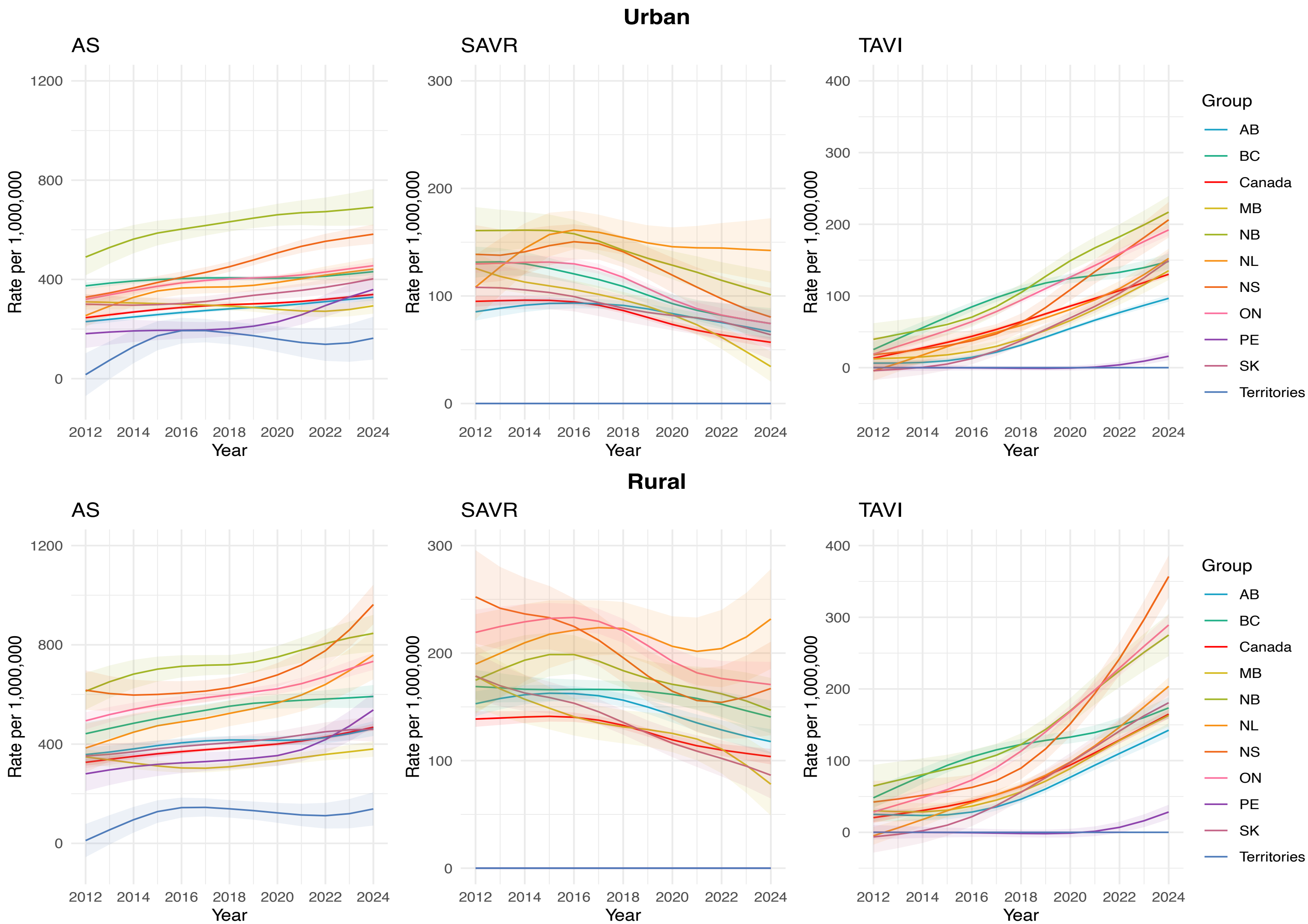

Supplement: Supplementary file 1 [file mmc1.pdf]
